# Radiation exposure of computed tomography imaging for the assessment of acute stroke

**DOI:** 10.1007/s00234-020-02548-z

**Published:** 2020-09-08

**Authors:** Sebastian Zensen, Nika Guberina, Marcel Opitz, Martin Köhrmann, Cornelius Deuschl, Michael Forsting, Axel Wetter, Denise Bos

**Affiliations:** 1 Department of Diagnostic and Interventional Radiology and Neuroradiology, University Hospital Essen, Hufelandstraße 55, 45147 Essen, Germany; 2 Department of Radiotherapy, University Hospital Essen, Hufelandstraße 55, Essen, 45147 Germany; 3 Department of Neurology, University Hospital Essen, Hufelandstraße 55, Essen, 45147 Germany

**Keywords:** Computed tomography perfusion, Radiation exposure, Stroke

## Abstract

**Purpose:**

To assess suspected acute stroke, the computed tomography (CT) protocol contains a non-contrast CT (NCCT), a CT angiography (CTA), and a CT perfusion (CTP). Due to assumably high radiation doses of the complete protocol, the aim of this study is to examine radiation exposure and to establish diagnostic reference levels (DRLs).

**Methods:**

In this retrospective study, dose data of 921 patients with initial CT imaging for suspected acute stroke and dose monitoring with a DICOM header–based tracking and monitoring software were analyzed. Between June 2017 and January 2020, 1655 CT scans were included, which were performed on three different modern multi-slice CT scanners, including 921 NCCT, 465 CTA, and 269 CTP scans. Radiation exposure was reported for CT dose index (CTDI_vol_) and dose-length product (DLP). DRLs were set at the 75th percentile of dose distribution.

**Results:**

DRLs were assessed for each step (CTDI_vol_/DLP): NCCT 33.9 mGy/527.8 mGy cm and CTA 13.7 mGy/478.3 mGy cm. Radiation exposure of CTP was invariable and depended on CT device and its protocol settings with CTDI_vol_ 124.9–258.2 mGy and DLP 1852.6–3044.3 mGy cm.

**Conclusion:**

Performing complementary CT techniques such as CTA and CTP for the assessment of acute stroke increases total radiation exposure. Hence, the revised DRLs for the complete protocol are required, where our local DRLs may help as benchmarks.

## Introduction

Stroke is a frequent cause of disability and death in adults [1] and the etiology of stroke is either ischemic or hemorrhagic [2]. Ischemic stroke accounts for more than 85% of acute strokes and has a high global morbidity and mortality [3, 4]. Due to rapid irreversible damage of tissue, quick and accurate diagnosis is crucial to enable prompt therapy [5, 6].

Non-contrast computed tomography (NCCT) of the head is widely recommended as initial imaging modality for suspected acute stroke because it is highly sensitive for depiction of intracranial hemorrhage [7, 8]. Moreover, NCCT can reveal early signs of infarction, although they occur only in a small proportion of patients [8, 9]. Computed tomography angiography (CTA) enables assessment of brain supplying extra- and intracranial vessels and provides additional information about occlusion site and origin of infarction [2, 10, 11]. Computed tomography perfusion (CTP) depicts cerebral hemodynamics and enables evaluation of vessel occlusion found during CTA [12, 13]. Since its establishment in the late 1990s, CTP expanded the value of CT techniques in acute stroke imaging because it detects irreversibly ischemic tissue and enables differentiation between irreversibly infarcted and ischemic but potentially salvageable tissue [2, 6, 14, 15].

A comprehensive CT protocol provides additional information and hereby improves the detection rate of ischemic stroke [2, 9, 16–18]. Alongside these benefits, CT is considered a high-dose imaging technique since its establishment and causes the major part of collective effective dose for all radiographic examinations and even increased within the last years [19–22].

Different national and multinational guidelines were published to limit radiation exposure and state diagnostic reference levels (DRLs) [23]. Hence, to monitor and optimize radiation exposure, standardized dose values need to be registered. The volume-weighted CT dose index (CTDI_vol_) quantifies the intensity of CT radiation that a patient is exposed to [24, 25]. The dose-length product (DLP) is the product of scan length and CTDI_vol_ and quantifies the total amount of ionizing radiation [26, 27]. Modern CT scanners display and archive technical dose descriptors such as CTDI_vol_ and DLP which are determined in polymethyl methacrylate (PMMA) phantoms of 16 or 32 cm in diameter [28, 29]. Consequently, as CTDI_vol_ and DLP quantify the radiation dose output of a CT scanner, they are appropriate reference parameters. Although they do not directly represent the dose to an individual patient, CTDI_vol_ and DLP might help to ensure lower radiation exposures [30]. To evaluate local radiation exposures, the 75th percentile of dose metric distributions is often used as DRL [31, 32]. Achievable dose (AD) is another useful benchmark and represents the 50th percentile of a distribution of ionizing radiation [32].

The aim of this study was to assess the distribution of radiation exposure of the CT protocol for the assessment of acute stroke and to establish local DRLs.

## Material and methods

### Patient cohort

Between June 2017 and January 2020, 2897 patients with acute stroke were treated at our center. Thereof, 921 patients who were identified using a DICOM header–based tracking and monitoring software (Radimetrics Enterprise Platform, Bayer Healthcare, Leverkusen, Germany) and who underwent initial CT examination for suspected acute stroke without prior imaging were included in this retrospective study. Dose data comprised 1655 CT scans using a local stroke protocol, consisting of 921 NCCT, 465 CTA, and 269 CTP examinations. Three subgroups could be determined: patients who underwent (I) NCCT solely, (II) NCCT and CTA, and (III) NCCT, CTA, and CTP. Ethical approval for this study was granted by the internal ethical committee of our institution and the requirement to obtain informed consent was waived (20-9175-BO).

### CT protocol and scanner

A standardized local “master” CT protocol for suspected stroke was used including initial NCCT, optional CTA from aortic arch to vertex, and optional CTP of the brain performed as implemented by the vendor. Clinical information and image-based findings were extracted from the report archived in the radiological information system (RIS). All scans were performed at one of three commercially available, modern multi-slice CT scanners: one single-source 128-slice SOMATOM Definition AS+, one dual-source 128-slice SOMATOM Definition Flash, and one dual-source 192-slice SOMATOM Force (all Siemens Healthcare, Forchheim, Germany). Technical settings according to device are shown in Table 1.

**Table 1:** Technical parameters of each step of the acute stroke CT protocol including non-contrast CT (NCCT) of the head, CT angiography (CTA), and CT perfusion (CTP) at three different multi-slice Siemens CT scanners

	NCCT			CTA			CTP		
Device*	AS+	Flash	Force	AS+	Flash	Force	AS+	Flash	Force
Dual-source mode	N/A	On	Off	N/A	On	Off	N/A	Off	Off
Pitch	0.55	0.7	0.55	0.9	0.45	0.35	0.45	0.5	0.5
Rotation time (s)	1	0.5	1	0.3	0.33	0.25	0.6	0.57	0.5
Tube voltage (kV)	100	80/Sn140^1^	100	80-120	80 / Sn140^1^	70–120	80	80	70
Tube current modulation	On	On	On	On	On	On	Off	Off	Off
Reference tube current-time product (mAs)	400	400/200	406	135–202	222/111	117–200	200	200	170
Slice thickness (mm)	0.6	0.6	0.6	0.6	0.6	0.6	1.2	1.2	1.2

*AS+: SOMATOM Definition AS+; Flash: SOMATOM Definition Flash; Force: SOMATOM Force (all: Siemens Healthcare, Forchheim, Germany)

^1^At tube B tin (Sn) filtration was applied

### Dose assessment

For dose assessment, the commercially available automated dose-tracking software Radimetrics Enterprise Platform based on Monte Carlo simulation techniques was used. Examination data and dose measurements were extracted from this software, which collects radiation exposure metadata and patient demographic information from the Digital Imaging and Communications in Medicine (DICOM) header and from the radiation dose structured report stored in the picture archiving and communication system (PACS) [33]. If dose modulation was used, the CT device took the variation in dose into account when calculating radiation parameters. Topogram- and monitoring-based radiation exposure data were excluded. Dose assessments referred to the 16-cm diameter standard head phantom for NCCT and CTP and to the 32-cm diameter body phantom for CTA.

### Statistics and data analysis

Descriptive statistics were performed using the GraphPad Prism 5.01 (GraphPad Software, San Diego, USA). DRLs were set at the 75th percentile and AD at the 50th percentile of dose distribution. To determine normal distribution, Kolmogorov-Smirnov and Shapiro-Wilk-test were applied. The examined variables did not follow normal distribution. The Kruskal-Wallis test with Dunn-Bonferroni post hoc test was applied to compare radiation doses in terms of CTDI_vol_ and DLP at the three different CT scanners. A *p*-value lower than 0.05 was considered statistically significant.

## Results

### Patient cohort

In our retrospective study, 921 patients underwent the acute stroke CT protocol at three different multi-slice CT scanners between June 2017 and January 2020. The mean age was 66.7 years ± 16.7 (SD) with a range from 18 to 101 years. About 62% of all examinations were performed at SOMATOM Force, 24.7% at Definition Flash, and 13.3% at Definition AS+ (Tables 2 and 3).

**Table 2:** Volume-weighted CT dose index (CTDI_vol_) of non-contrast CT (NCCT) of the head, CT angiography (CTA), and CT perfusion (CTP) for the assessment of acute stroke

			CTDI_vol_ [mGy]			
	Device	No. of scans	Median	IQR	Mean ± SD	DRL
NCCT^1^	Total	921	31.4	28.9–33.9	31.5 ± 3.8	33.9
	AS+	107	29.7	28.5–31.4	30.2 ± 3.9	31.4
	Flash	163	27.0	25.8–28.9	27.6 ± 3.1	28.9
	Force	651	32.4	30.2–34.6	32.6 ± 3.2	34.6
CTA^2^	Total	465	9.3	6.0–13.7	10.0 ± 4.3	13.7
	AS+	75	5.8	5.4–6.0	5.9 ± 1.4	6.0
	Flash	146	14.5	13.2–15.6	14.2 ± 2.0	15.6
	Force	244	8.6	6.2–9.8	8.8 ± 3.8	9.8
CTP^1, 3^	Total	269	219.8	124.9–258.2	187.9 ± 62.7	258.2
	AS+	38	219.8	219.8–219.8	219.8 ± 0.2	219.8
	Flash	100	258.2	258.2–258.2	258.2 ± 0.0	258.2
	Force	131	124.9	124.9–124.9	124.9 ± 0.1	124.9

AS+: SOMATOM Definition AS+; Flash: SOMATOM Definition Flash; Force: SOMATOM Force (all: Siemens Healthcare, Forchheim, Germany)

^1^CTDI_vol_ refers to the 16-cm polymethyl methacrylate (PMMA) phantom

^2^CTDI_vol_ refers to the 32-cm polymethyl methacrylate (PMMA) phantom

^3^For CTP, each slice was scanned 30 times

**Table 3:** Dose-length product (DLP) of non-contrast CT (NCCT) of the head, CT angiography (CTA), and CT perfusion (CTP) for the assessment of acute stroke

			DLP [mGy cm]			
	Device*	No. of scans	Median	IQR	Mean ± SD	DRL
NCCT	Total	921	480.9	436.5–527.8	486.6 ± 72.5	527.8
	AS+	107	457.4	430.4–497.6	476.1 ± 85.7	497.6
	Flash	163	429.3	397.5–463.2	434.1 ± 54.5	463.2
	Force	651	495.7	451.6–543.1	501.5 ± 67.6	543.1
CTA	Total	465	329.5	224.6–478.3	359.4 ± 159.4	478.3
	AS+	75	212.4	199.6–227.4	222.9 ± 69.2	227.4
	Flash	146	510.7	446.9–563.8	506.7 ± 89.3	563.8
	Force	244	298.2	218.0–355.0	313.3 ± 146.2	355.0
CTP	Total	269	2504.0	1853.0–3044.0	2388.0 ± 550.2	3044.0
	AS+	38	2504.0	2504.0–2504.0	2504.0 ± 2.1	2504.0
	Flash	100	3044.0	3044.0–3044.0	3044.0 ± 0.0	3044.0
	Force	131	1853.0	1853.0–1853.0	1853.0 ± 0.1	1853.0

*AS+: SOMATOM Definition AS+; Flash: SOMATOM Definition Flash; Force: SOMATOM Force (all: Siemens Healthcare, Forchheim, Germany)

### Radiation exposures of each step of the CT protocol

Dose assessment comprised radiation exposure data of 1655 CT scans including 921 NCCT, 465 CTA, and 269 CTP examinations. The mean radiation exposure in terms of CTDI_vol_ and DLP was distributed as follows median (interquartile range; mean ± SD): for NCCT, 31.4 mGy (28.9–33.9; 31.5 ± 3.8) and 480.9 mGy cm (436.5–527.8; 486.6 ± 72.5), and for CTA, 9.3 mGy (6.0–13.7; 10.0 ± 4.3) and 329.5 mGy cm (224.6–478.3; 359.4 ± 159.4). CTP radiation exposure is invariable and depends on CT device as well as its protocol settings: (CTDI_vol_, DLP) for SOMATOM Definition AS+, 219.8 mGy and 2503.9 mGy cm; for SOMATOM Definition Flash, 258.2 mGy and 3044.3 mGy cm; and for SOMATOM Force, 124.9 mGy and 1852.6 mGy cm. The detailed results differentiated by the device are shown for CTDI_vol_ in Table 2 and for DLP in Table 3.

### Local DRLs and ADs of the CT protocol

Local DRLs in terms of CTDI_vol_ and DLP could be depicted as follows (versus national DRLs proposed by the Federal Office for Radiation Protection) (Bundesamt für Strahlenschutz, Germany [34]): for NCCT, 33.9 mGy (60 mGy) and 527.8 mGy cm (860 mGy cm), and for CTA, 13.7 mGy (20 mGy) and 478.3 mGy cm (600 mGy cm). ADs could be determined as follows: (CTDI_vol_, DLP) for NCCT, 31.4 mGy and 480.9 mGy cm and for CTA, 9.3 mGy and 329.5 mGy cm. As radiation indices of CTP were uniform and scanner-specific, we waived to determine DRL and AD.

### Comparison between CT scanners

In terms of CTDI_vol_, NCCT showed very similar distribution of radiation exposure between the investigated CT scanners with the lowest CTDI_vol_ at SOMATOM Definition Flash (median 27.0 mGy, IQR 25.8–28.9 mGy), highest at SOMATOM Force (median 32.4 mGy, IQR 30.2–34.6 mGy), and SOMATOM Definition AS+ in between (median 29.7 mGy, IQR 28.5–31.4 mGy) (Table 2). In comparison, the radiation exposures of CTA varied substantially: the highest dose was required by SOMATOM Definition Flash (median 14.5 mGy, IQR 13.2–15.6 mGy) and lowest by SOMATOM Definition AS+ (median 5.8 mGy, IQR 5.4–6.0 mGy). CTDI_vol_ for CTA at SOMATOM Force was in between (median 8.6 mGy, IQR 6.2–9.8 mGy). Accordingly, median CTDI_vol_ was about 2.5 times higher (2.4 for DLP) at SOMATOM Definition Flash and about 1.5 times higher (1.4 for DLP) at SOMATOM Force compared with SOMATOM Definition AS+. The Kruskal-Wallis test with Dunn-Bonferroni post hoc test revealed that CTDI_vol_ and DLP for NCCT and CTA were significantly different between the three different CT scanners (*p < 0.001*). Concerning CTP, the lowest CTDI_vol_ was achieved by SOMATOM Force (median 124.9 mGy) which was about 50% lower than SOMATOM Definition Flash (median 258.2 mGy). Radiation exposure at SOMATOM Definition AS+ was in between and slightly lower than SOMATOM Definition Flash (median 219.8 mGy). Differences between the three CT scanners were similar in terms of DLP compared with CTDI_vol_ (Table 3).

## Discussion

Our study reveals useful radiation exposure data and local DRLs for a multimodal stroke CT protocol and shows that modern multi-slice CT scanners enable imaging with lower radiation exposure than national DRLs. However, the multimodal CT protocol increases total radiation exposure significantly, which must be balanced with diagnostic benefits.

### Radiation exposure

Monitoring radiation exposure of CT examinations helps to ensure radiation protection and provides essential information to optimize CT protocols [35]. Therefore, standardized collecting of radiation dose data is a common practice at our institute. Our results are in keeping with previous studies which reported that a multimodal CT protocol requires relative high radiation exposure [4]. Accordingly, CT procedures are considered to comprise stochastic radiation risks [36]. Nevertheless, CTDI_vol_ and DLP are not intended to derive estimates of individual patient risk as they do not include radiosensitivity of organs [27, 37]. However, CTDI_vol_ and DLP are useful values which measure the ionizing radiation emitted from the scanner and therefore act as quality control metrics, which represent exposure values rather than patient doses [35]. DRLs are helpful benchmark which indicate typical ionizing radiation exposure values in a country, region, or an institute [38]. Several studies have reported local, regional, or national DRLs which is a reasonable solution as equipment and protocols vary by institutes and, a fortiori, by nations [38].

### Comparison with national and published local DRLs

**Fig. 1:**
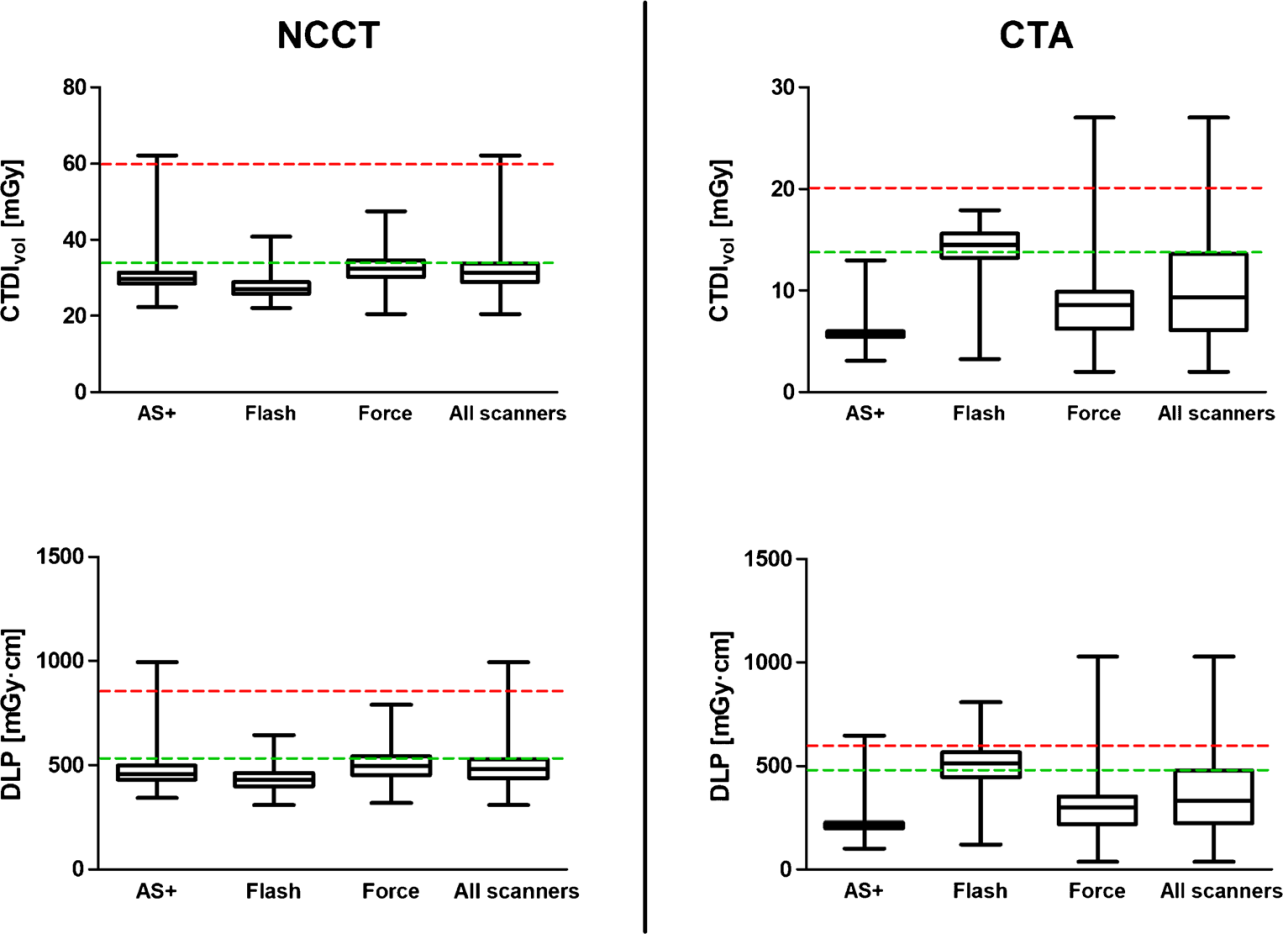
Distribution of radiation exposure for volume-weighted CT dose index (CTDI_vol_) (mGy) and dose-length product (DLP) (mGy cm) of non-contrast CT (NCCT) of the head and CT angiography (CTA) for the assessment of acute stroke. Radiation exposure of three different multi-slice CT scanners (SOMATOM Definition AS+, Definition Flash, and Force; Siemens Healthcare, Forchheim, Germany). Red broken line represents national dose reference level (DRL) for NCCT, CTDI_vol_ 60 mGy and DLP 850 mGy cm, and for CTA, CTDI_vol_ 20 mGy and DLP 600 mGy cm. Green broken line represents determined local DRL which is well below the national DRL. Whiskers represent min to max

German national DRL for NCCT is set at 60 mGy (CTDI_vol_) and 850 mGy cm (DLP) [34]. DRLs at our institution for NCCT and CTA were below national DRLs (Fig. 1). The highest CTA DLP was 1028 mGy cm with a CTDI_vol_ of 26.4 mGy, concluding that the major part of radiation excess was due to expanded scan length. However, national DRLs exist for NCCT and CTA only and there are no DRLs for CTP for the assessment of stroke. Further, DRLs for NCCT are set by the European Commission: weighted CT dose index (CTDI_w_) 60 mGy and DLP 1050 mGy cm [23]. In comparison with previous studies from different European countries, our local values are remarkable below the published DRLs. Most DRLs for NCCT vary between 60 and 80 mGy (CTDI_vol_) [39–41]. Besides, we calculated ADs as another reference value to encourage further optimization of radiation protection [42]. The whole gamut of our radiation exposure is well below the reported data: exposures are below several recently reviewed studies reporting NCCT DRLs from different countries with a DLP ranging from 787 to 1305 mGy cm [35] and a median DLP of 733 mGy cm with a range from 81 to 2173 mGy cm [37]. Ionizing radiation exposure of CT can vary significantly by institution [43, 44]. In addition, our results correspond with previous studies that demonstrated variation of radiation exposure due to intrinsic differences of CT scanners [45, 46]. Mnyusiwalla et al. reported a mean radiation exposure six times higher for a multimodal CT protocol for acute stroke compared with NCCT solely [4]. Such high radiation doses must be balanced with the benefits of enhanced anatomic and prognostic data [4].

### Comparison between CT scanners

The lowest radiation exposure for NCCT in terms of CTDI_vol_ was depicted at SOMATOM Definition Flash, even though it was the only scanner where NCCT was acquired in dual-source mode (Tables 1 and 2). This can be affiliated to the increased pitch and decreased rotation time, which both result in decreased radiation dose. Nonetheless, radiation exposure indices and DRLs showed similar distributions at all three investigated CT scanners. For CTA, substantially diverging radiation exposures were depicted at the three CT scanners. Application of dual-source mode at SOMATOM Definition Flash and different technical settings resulted in substantially higher radiation dose compared with SOMATOM Definition AS+ and Force (Table 2). Variation of pitch as an inversely proportional parameter and of rotation time as a directly proportional parameter to CTDI_vol_ caused the radiation exposures to differ considerably. Our results demonstrate that radiation exposure from CTP is invariable and depends on CT device as well as its protocol settings. Nonetheless, several studies have reported modifications to reduce radiation exposure of CTP [17, 47]. Furthermore, Othman et al. demonstrated that low-dose CTP generates sufficient perfusion maps [48]. In conclusion, if institutes perform CTP examinations as recommended by the vendor, radiation exposure is virtually a CT scanner-specific parameter, and modifying protocols will not reduce the proportion of radiation by CTP unless scanner settings are modified. Our data show that radiation exposure of CTP can vary substantially. Tube current-time product, tube voltage, and rotation time were decreased at SOMATOM Force which resulted in the lowest CTDI_vol_ compared with SOMATOM Definition AS+ and Definition Flash.

### Radiation risks

While short-term effects of ionizing radiation from CT techniques for stroke are rare, long-term effects are not entirely known [4]. Imanishi et al. reported three cases of bandage-shaped scalp hair loss in patients undergoing serial CTP studies combined with digital subtraction angiography, although it should be mentioned that these patients received multiple examinations [49]. Regarding long-term effects, the estimate for risk of fatal cancer due to radiation exposure is 5% per Sievert [50] but excess cancer mortality also depends on patients’ age and attributable risk of cancer decreases with age [51]. However, full CT protocol was indicated due to strong clinical suspicion of acute stroke. Fortunately, the major proportion of our patients carried a lower risk of radiation exposure–related cancer mortality due to a mean age of 66.7 ± 16.7 years (SD). Nevertheless, we strongly advocate a judicious use of a multimodal CT protocol, in particular for young patients due to their heightened risk from ionizing radiation [4].

### Acute stroke CT protocol

As widely recommended [7], we use NCCT as the initial imaging modality in acute stroke to exclude intracranial hemorrhage or stroke mimicking etiologies. As inconspicuous initial NCCT examinations cannot be equated with exclusion of ischemic stroke, further assessment is required in a major proportion of patients. Therefore, there is an increasing interest in advanced and complementary CT techniques for the evaluation of acute stroke [2, 6]. Possible detection of occluded extra- and intracranial arteries provides an argument for performing a subsequent CTA [2, 10, 11]. Furthermore, complementary CTA and CTP provide additional, often relevant, information [2, 52–54]. Moreover, in agreement with the findings in previous studies [2, 55], we confirm that a multimodal CT protocol can be performed as a single-time examination and takes less than 10 min more than NCCT only.

### Advantages and limitations

About 62% of all CT scans were performed at SOMATOM Force which results in an underrepresentation of the other CT devices. Only patients who were identified by the DICOM header–based tracking and monitoring software were included in this study, which can result in a sampling bias. The strengths of our study include the large number of patients assessed by a uniform CT protocol and the detailed report of radiation exposure between different modern multi-slice CT scanners.

## Conclusion

Performing complementary advanced CT techniques such as CTA and CTP for the assessment of acute stroke increases total radiation exposure substantially. Hence, revised and completed DRLs for the acute stroke CT protocol are required, where our locally examined DRLs may help as benchmarks as they were well below the major proportion of recently published DRLs. DRLs regarding a full CT imaging workup for acute stroke assessment enable optimization of local radiation protection and provide actual reference values for dose monitoring.

## Abbreviations

AD: Achievable dose
NCCT: Non-contrast computed tomography
CTA: Computed tomography angiography
CTDI_vol_: Volume-weighted CT dose index
CTDI_w_: Weighted CT dose index
CTP: Computed tomography perfusion
DLP: Dose-length product
DRL: Diagnostic reference level
IQR: Interquartile range
kV: Kilovolt (tube voltage)
mAs: Milliampere second (tube current-time product)
RIS: Radiological information system
SD: Standard deviation
